# Preserving Patient Influence During Interrupted Guideline Participation

**DOI:** 10.1002/gin2.70093

**Published:** 2026-09-28

**Authors:** Ting‐Yi Hung, Lien‐Chung Wei

**Affiliations:** ^1^ Department of General Psychiatry, Taoyuan Psychiatric Center Ministry of Health and Welfare Taoyuan City Taiwan; ^2^ Department of Addiction Psychiatry, Taoyuan Psychiatric Center Ministry of Health and Welfare Taoyuan City Taiwan; ^3^ Department of Health Industry Management Kainan University Taoyuan City Taiwan; ^4^ Department of Counseling and Industrial/Organizational Psychology Ming Chuan University – Taoyuan Campus Taoyuan City Taiwan

To the Editor,

1

Orduhan and colleagues show how training, supportive group dynamics and recognition can strengthen patient involvement in guideline development [1]. They appropriately acknowledge the educational and organisational selectivity of their sample. A further question is whether participation remains influential when it cannot remain continuous. The study recruited people with previous guideline experience and did not collect reasons for nonparticipation [1]. Its findings therefore do not directly evaluate support for contributors whose availability changes during guideline development.

This distinction matters when translating the findings to mental health guidelines. A person's expertise by experience does not disappear during a period of reduced availability. Nor should a psychiatric diagnosis be taken as evidence that participation will be unreliable. The relevant design question is whether the process accommodates individually identified, changing support needs. The GIN toolkit already recommends reassessing support because physical and mental health conditions can fluctuate, alongside adjustments to meetings and communication [2]. These existing recommendations provide a foundation for evaluating whether flexibility preserves influence.

We propose evaluating participation trajectories, not only the experiences of established panel members. With proportionate consent and privacy safeguards, developers could record invitations, acceptance, temporary pauses, re‐entry and chosen contribution formats. Reasons for declining or pausing should be optional; collecting these data should not require disclosure of a psychiatric diagnosis. These observations would describe where access is lost, without treating every absence as disengagement or claiming that a small panel statistically represents all affected people.

Before finalising a recommendation, panels could offer a time‐limited opportunity for written, recorded or supported verbal input from members unable to attend the relevant meeting. An agreed return process should explain decisions already made and identify decisions still open to influence. A brief contribution‐to‐decision record could show whether an issue changed the scope, outcome selection, recommendation wording, or implementation advice, or explain why no change followed. This is not a requirement to accept every suggestion. It makes consideration visible without equating continuous attendance with influence. Reporting should minimise identifiable information and avoid turning support into attendance surveillance.

A parallel‐group guideline study found differences in patient‐relevant topics and outcomes despite substantial overlap between groups' proposed questions [3]. The WHO meaningful‐engagement framework likewise advocates co‐creating policies and services with people with lived experience [4]. Together, these perspectives support a testable extension of Orduhan and colleagues' findings: assess whether flexible participation preserves substantive influence, while measuring participant burden as well as retention. A successful process should permit a pause without silently converting a contributor into an observer.

## Author Contributions

**Ting‐Yi Hung:** conceptualisation, writing – review and editing (critical intellectual review). **Lien‐Chung Wei:** conceptualisation, literature review, writing – original draft, writing – review and editing. Both authors approved the final manuscript and agree to be accountable for all aspects of the work.

## Funding

The authors have nothing to report.

## Ethics Statement

Ethics approval is not applicable as this letter reports no new research involving human participants or animals and presents no new data.

## Consent

Participant consent is not applicable as this letter reports no new research involving human participants or animals and presents no new data.

## Conflicts of Interest

The authors declare no conflicts of interest.

## Data Availability

Data sharing is not applicable because no new data sets were generated or analysed.
